# Supplementary material for: Tuberculosis treatment outcomes and associated factors at Alemgena Health Center, Sebeta, Oromia, Ethiopia
Source: PLoS One. 2024 May 21;19(5):e0303797. doi: 10.1371/journal.pone.0303797 (PMC11108144; doi:10.1371/journal.pone.0303797)
Supplement: Attachment — Submitted filename: Response to Reviewers.docx [file pone.0303797.s002.docx]

Date: March 04, 2024

Dear Prof. Frederick Quinn,

I thank you so much for your comments and consideration. I am also grateful to the reviewers for proving comments that led to the improvement of the quality of our manuscript. I am sorry for not meeting the deadline for a reason beyond my control.

I have responded to all comments of the academic editor and that of the reviewers.

The responses (in green) are typed below each comment.

I look forward to hearing from you about your decision on the revised manuscript.

Kind regards,

Dagim Jirata Birri

Corresponding author

**Response Academic Editor and Reviewers**

Journal Requirements:

1. When submitting your revision, we need you to address these additional requirements.

Please ensure that your manuscript meets PLOS ONE's style requirements, including those for file naming. The PLOS ONE style templates can be found at 
<https://journals.plos.org/plosone/s/file?id=wjVg/PLOSOne_formatting_sample_main_body.pdf>

and 
<https://journals.plos.org/plosone/s/file?id=ba62/PLOSOne_formatting_sample_title_authors_affiliations.pdf>

Response: The title page and the main body of our manuscript is formatted based on the recommended templates.

2. In the online submission form, you indicated that Data will be obtained from the corresponding author up on request.

All PLOS journals now require all data underlying the findings described in their manuscript to be freely available to other researchers, either 1. In a public repository, 2. Within the manuscript itself, or 3. Uploaded as supplementary information.
This policy applies to all data except where public deposition would breach compliance with the protocol approved by your research ethics board. If your data cannot be made publicly available for ethical or legal reasons (e.g., public availability would compromise patient privacy), please explain your reasons on resubmission and your exemption request will be escalated for approval.

Response: I have uploaded data as supplementary information.

3. Please amend your list of authors on the manuscript to ensure that each author is linked to an affiliation. Authors’ affiliations should reflect the institution where the work was done (if authors moved subsequently, you can also list the new affiliation stating “current affiliation:….” as necessary).

 Response: I have amended the list of authors on the manuscript.

[Note: HTML markup is below. Please do not edit.]

Reviewers' comments:

Reviewer's Responses to Questions

**Comments to the Author**

1. Is the manuscript technically sound, and do the data support the conclusions?

The manuscript must describe a technically sound piece of scientific research with data that supports the conclusions. Experiments must have been conducted rigorously, with appropriate controls, replication, and sample sizes. The conclusions must be drawn appropriately based on the data presented.

Reviewer #1: Partly

Response: Our manuscript is based on secondary data, not on experimentally generated data. So comments about control and replication are not applicable here. The sample size was large and enough and our conclusions are based on the data presented in the manuscript.

Reviewer #2: Yes

2. Has the statistical analysis been performed appropriately and rigorously?

Reviewer #1: No

Response: This comment is not clear since it does not specify what kind specific statistical analysis is needed, we believe that appropriate statistical analysis was done.

Reviewer #2: Yes

3. Have the authors made all data underlying the findings in their manuscript fully available?

The [PLOS Data policy](http://www.plosone.org/static/policies.action#sharing) requires authors to make all data underlying the findings described in their manuscript fully available without restriction, with rare exception (please refer to the Data Availability Statement in the manuscript PDF file). The data should be provided as part of the manuscript or its supporting information, or deposited to a public repository. For example, in addition to summary statistics, the data points behind means, medians and variance measures should be available. If there are restrictions on publicly sharing data—e.g. participant privacy or use of data from a third party—those must be specified.

Reviewer #1: Yes

Reviewer #2: Yes

4. Is the manuscript presented in an intelligible fashion and written in standard English?

PLOS ONE does not copyedit accepted manuscripts, so the language in submitted articles must be clear, correct, and unambiguous. Any typographical or grammatical errors should be corrected at revision, so please note any specific errors here.

Reviewer #1: No

Response: We have corrected language errors.

Reviewer #2: No

Response: We have corrected language errors.

5. Review Comments to the Author

Please use the space provided to explain your answers to the questions above. You may also include additional comments for the author, including concerns about dual publication, research ethics, or publication ethics. (Please upload your review as an attachment if it exceeds 20,000 characters)

Reviewer #1:

**General comments**: This manuscript has generated important data for TB control program in local context. However, it has limited global implication. If the authors re-write the manuscript including re-analysis it can be considered for publishing in Plos One.
Detail comments as follow;

Response: We have re-written our manuscript.

**Abstract**
Result: Line 16-19: Please, avoid ambiguous words like about, nearly, almost all. Such words are frequently used even in the body of manuscript

**Response:** Ambiguous words were removed the entire manuscript.

**Introduction**:
Line 31-32: ---------. Humans are the only
reservoir for MTB. There are number recent report on MTB infections in animals how authors dare to say human are the only reservoir for MTB? Can authors justify?

Response: The sentence was re-written (Humans are the only natural reservoir for MTB). and reference cited.
Line 30-38: whole paragraph has no reference citation

Response: References are cited
Authors used almost same source of information, WHO report and advised to use scientific report/journal articles rather than using report.

Response: We used WHO report since WHO is the only organization responsible for gathering epidemiological data on health problems, including TB, from member countries and publishing data.

Moreover, authors used different citation style in one paper. For instance, author, date (WHO, 2021) and number in bracket (2). Authors should use Plos One reference style

Response: Citations were correct to be consistent and are according to Plos One style.

**Materials and Methods**
Study design: Line 109: typo error . A 5 years………..was employed to to achieve……

Response: corrected
Line: 111-121: Authors advised to organize under one sub-heading.
Response: Organized under one sub-heading (study design, population and sampling)

Ethical consideration
Although authors obtained ethical approval for study form ethical review board of Ayer Tena Health Science and Business College, they didn’t mention about consent of Alemgena health centre to give them permission to use their data for this study.

Response: Mentioned

**Results**: Line 168-169: (standard deviation = 14.71). Nearly 90% (889/990) of the patients were in the age group 0-50----. Age can be <1 year, but how can be zero?

Response: The grouping of age was corrected in such a way that it excludes zero.
Line 181-182: Data on patient category, smear result, TB type and HIV status were not
available for 82, 381, 27 and 67 patients, respectively. What are the numbers 82, 381, 27 and 67?

Response: Made clear to show that these numbers represent the number of TB patients for which respective data were not available.

Table 1: Line 188: what is the reason for categorizing transfer in together with new and relapse case? New and relapse can be linked to DRTB and transmission trend and transfer in link to what?

Response: That is how Health Ministry of Ethiopia classifies TB patients. It is found in the TB patients register. The same classification was used in many published studies, including those from Ethiopia and other countries. Some are listed below:

<http://dx.doi.org/10.1155/2016/1354356>

<https://doi.org/10.1186/s13104-017-3020-z>

<https://doi.org/10.1371/journal.pone.0205468>

doi: 10.3389/fmed.2018.00038

DOI: 10.1177/0300060520984916

https://d oi.org/10.1155/2021/9952806

doi:10.3855/jidc.5963

<https://doi.org/10.1016/j.jiph.2019.10.012>

Reviewer #2:

1. Paragraph one lacks references

Response: References are cited now.
2. Reference system is mixed one another citations in side the body of the manuscript are not uniform (on the following line numbers: L44,45,46, 49,51,54, 56,58,60,62,65,83 the authors used text citations inside in the other part they used numbering system).

Response: All citations were made consistent (numbering style).
3. Line 87-89... the author used old version reference and it is advisable to put the recent updated reference instead.

Response: The most recent reference is cited.
4. Instead of a general reference to a 5-year retrospective study, it would be clearer to specify the exact time frame, such as ‘Institutional based retrospective study on a data collected from September 2012 to August 2018.' This removes any ambiguity and gives readers a precise understanding of the data analyzed as the study used specific health center data only.

Response: Corrected by specifying the time period September 2012 to August 2018.
5. Line number 136-138: in line 137 its advisable to check the grammar and put ‘used’ and ‘and’ next to ‘was’ and ‘outcomes’.

Response: Corrected by inserting ‘used’

6. The author used old references and its recommended to use recent edition.

Response: Recent references were included.
